# Molecular pathways undergoing dramatic transcriptomic changes during tumor development in the human colon

**DOI:** 10.1186/1471-2407-12-608

**Published:** 2012-12-19

**Authors:** Rosalia Maglietta, Vania Cosma Liuzzi, Elisa Cattaneo, Endre Laczko, Ada Piepoli, Anna Panza, Massimo Carella, Orazio Palumbo, Teresa Staiano, Federico Buffoli, Angelo Andriulli, Giancarlo Marra, Nicola Ancona

**Affiliations:** 1 Istituto di Studi sui Sistemi Intelligenti per l'Automazione - C.N.R., Via Amendola 122/D-I, 70126 Bari, Italy; 2 Institute of Molecular Cancer Research, University of Zurich, Winterthurerstrasse 190, 8057, Zurich, Switzerland; 3 Functional Genomics Center, University of Zurich, Winterthurerstrasse 190, 8057, Zurich, Switzerland; 4 Divisione di Gastroenterologia IRCCS “Casa Sollievo della Sofferenza” Ospedale, Viale Cappuccini, 71013, San Giovanni Rotondo, (FG), Italy; 5 Servizio di Genetica Medica IRCCS “Casa Sollievo della Sofferenza” Ospedale, Viale Cappuccini, 71013, San Giovanni Rotondo, (FG), Italy; 6 Endoscopy and Gastroenterology Unit, Hospital of Cremona, Cremona, Italy

**Keywords:** Colorectal adenoma, Colorectal cancer, Transcriptomics, Molecular pathways, Cell cycle pathways, Random set method

## Abstract

**Background:**

The malignant transformation of precancerous colorectal lesions involves progressive alterations at both the molecular and morphologic levels, the latter consisting of increases in size and in the degree of cellular atypia. Analyzing preinvasive tumors of different sizes can therefore shed light on the sequence of these alterations.

**Methods:**

We used a molecular pathway-based approach to analyze transcriptomic profiles of 59 colorectal tumors representing early and late preinvasive stages and the invasive stage of tumorigenesis. Random set analysis was used to identify biological pathways enriched for genes differentially regulated in tumors (compared with 59 samples of normal mucosa).

**Results:**

Of the 880 canonical pathways we investigated, 112 displayed significant tumor-related upregulation or downregulation at one or more stages of tumorigenesis. This allowed us to distinguish between pathways whose dysregulation is probably necessary throughout tumorigenesis and those whose involvement specifically drives progression from one stage to the next. We were also able to pinpoint specific changes within each gene set that seem to play key roles at each transition. The early preinvasive stage was characterized by cell-cycle checkpoint activation triggered by DNA replication stress and dramatic downregulation of basic transmembrane signaling processes that maintain epithelial/stromal homeostasis in the normal mucosa. In late preinvasive lesions, there was also downregulation of signal transduction pathways (e.g., those mediated by G proteins and nuclear hormone receptors) involved in cell differentiation and upregulation of pathways governing nuclear envelope dynamics and the G2>M transition in the cell cycle. The main features of the invasive stage were activation of the G1>S transition in the cell cycle, upregulated expression of tumor-promoting microenvironmental factors, and profound dysregulation of metabolic pathways (e.g., increased aerobic glycolysis, downregulation of pathways that metabolize drugs and xenobiotics).

**Conclusions:**

Our analysis revealed specific pathways whose dysregulation might play a role in each transition of the transformation process. This is the first study in which such an approach has been used to gain further insights into colorectal tumorigenesis. Therefore, these data provide a launchpad for further exploration of the molecular characterization of colorectal tumorigenesis using systems biology approaches.

## Background

Colon carcinogenesis is a multistep process involving the gradual accumulation of genetic and epigenetic alterations. These changes promote the malignant transformation of precancerous lesions of the colorectal mucosa [1], a process reflected by progressively severe cellular dysplasia and increases in lesion size. At least two-thirds of all colorectal cancers develop from precancerous lesions with adenomatous features [2]. The “serrated” histotype characterized by cells arranged in a saw-toothed pattern [1] is somewhat less common, but in both cases, size is an important indicator of the distance the lesion has travelled on the road toward malignancy. For this reason, post-polypectomy surveillance guidelines vary depending in part on the size of the polyps removed. In fact, individuals with 3 or more adenomas on initial colonoscopy, including 1 or more measuring ≥10 mm, are significantly more likely to present with new lesions at the next colonoscopy [3].

Analysis of precancerous colorectal lesions of different sizes can thus furnish important information on the steps involved in their malignant transformation. During colonoscopy, benign lesions of all sizes are routinely removed to prevent their progression toward cancer, and this provides a valuable source of tissues for molecular studies. Efforts of this type have already identified several genetic and epigenetic changes that seem to occur at the transition from normal mucosa to precancerous lesions. Mutations involving the *APC* or *CTNNB1* gene, for example, are considered early events that fuel epithelial-cell proliferation [4,5]. Gain-of-function mutations in the oncogenes *KRAS* and *BRAF* are also frequent findings in early stages of transformation [6]. Additional alterations (genetic and epigenetic) are believed to be necessary for subsequent steps toward invasiveness, such as those identified with recent genome-wide analyses [7,8].

The transcriptomes of colorectal cancers have been intensively investigated with high-throughput, array-based tools, which furnish quantitative, genome-wide descriptions of the individual gene expression levels associated with different cell phenotypes (e.g., adenoma cells vs. normal epithelial cells) [9-12]. More recently, other methods of analyzing gene expression data have been developed to gain additional insight into the mechanisms driving the phenotypic differences. One such approach involves the analysis not of single genes but of predefined functional *gene sets*, that is, groups of genes that are known components of a defined molecular pathway representing a given biological process. The basic aim here is to identify those gene sets (i.e., pathways) that display *enrichment* for―or over-representation of― genes whose expression is substantially altered in the phenotype being investigated. We have explored several methods for quantitatively analyzing transcriptomic data for pathway enrichment [13-15], including gene set enrichment analysis (GSEA) [16], random-set methods (RS) [17], and gene list analysis with prediction accuracy (a method developed by our group) [15]. Although these methods differ substantially from one another, all three are statistically accurate and identify relevant gene sets, and none consistently outperforms the others [14].

Our experience indicates that pathway-based analysis of gene expression data furnishes highly reproducible results that can be useful for dissecting a complex, polygenic disease like colorectal cancer. For instance, we recently used GSEA and RS analysis to identify pathway enrichment in four independent transcriptional data sets representing colorectal cancer and normal mucosa. The results of these analyses displayed substantial overlap: both of the analytical methods used revealed similar dysregulation of 53 pathways in each of the four data sets. These pathways are very likely to play important roles in the pathology of colorectal cancer [13].

In the present study, we used RS analysis to explore a large body of previously collected transcriptomic data on human colorectal tissues, including normal mucosa, preinvasive lesions of various sizes, and colorectal cancers (CRCs). Our aim was to identify biological processes that become dysregulated during the course of colorectal tumorigenesis. Because the preinvasive stages have been far less extensively explored than the cancerous phases of this process, there were no independent sets of transcriptomic data on precancerous lesions that we could use to validate our findings. To overcome this limitation, we used two strategies. First, we re-analyzed all the original data sets with GSEA and compared the results with those obtained with RS. Second, we performed RS analysis of two publicly available sets of data on CRCs and normal colorectal mucosa.

## Methods

All data were analyzed in MatLab (MathWorks, Natick, MA) unless otherwise stated.

### Data set

The data set analyzed in this study consisted of the transcriptome profiles of a series of 118 human colorectal tissues (details below) analyzed with the GeneChip Human Exon 1.0 ST array (Affymetrix, Santa Clara, CA, USA). Raw microarray data are available in GEO (GSE21962 [18]) and ArrayExpress (E-MTAB-829).

In brief, arrays were analyzed in the Affymetrix GeneChip Scanner 3000 7 G. Cell intensities were measured with Affymetrix GeneChip Operating Software, and Affymetrix Expression Console Software was used for quality assessment: probe expression intensity in each tissue sample was subjected to background adjustment and normalization with the Robust Multi-array Analysis algorithm.

The tissues themselves had been prospectively collected during colonoscopy (precancerous lesions) or surgery (cancers). They consisted of 59 tumor specimens, each accompanied by a sample of normal mucosa collected in the same colon segment >2 cm from the lesion. The fragment used for microarray analysis (~20 mg of epithelial tissue) was cut from each specimen immediately after removal, leaving the underlying *muscularis mucosae* intact, and the remaining tissue was submitted for pathologic analysis. (We used only lesions measuring >1 cm to ensure that our sampling procedure would not interfere with the histologic diagnosis.) All tumors were sporadic lesions with a functional DNA mismatch repair system. As expected, LPLs were more likely to exhibit villous changes (43.5% vs. 36.8% of the SPLs) and high-grade dysplasia (34.8% vs. 10.5% of the SPLs).

For the purposes of the present study, we divided the gene expression data into four subsets representing successive stages of colorectal tumorigenesis: 19 *small preinvasive lesions* (SPLs) measuring 11–20 mm in diameter, 23 *large preinvasive lesions* (LPLs) with diameters > 20 mm, and 17 CRCs (Table 1). A fourth set was created with data for all 59 *normal mucosal* (N) samples. The 20-mm cutoff for SPLs was chosen in part to obtain two similarly sized subgroups (for statistical purposes) and in part because our previous observations [18] suggested such subgroups are likely to present biological differences. All of the preinvasive lesions were adenomas except five, which exhibited serrated histology. These five lesions were included since they did not behave as outliers in Principal Component Analysis (PCA), and their exclusion did not significant alter the data reported in this study.

**Table 1:** Characteristics of the 59 colorectal tumors included in the study data set

**Preinvasive lesions**						
**Patient**	**Age**	**Sex**	**Colon segment**	**Diameter (mm)**	**Stage**^**†**^	**Histologic grade**^**‡**^
**Early stage (SPLs, diameters: 11–20 mm)**						
13b *	54	F	Ascending	12	IIa	TA (low)
21	66	M	Ascending	12	IIa	MVSP (none)
27	83	M	Sigmoid	12	Ip	TVA (low)
8	48	M	Hepatic flexure	15	IIa	TA (low)
15	62	F	Ascending	15	IIa	SA (low)
16	52	F	Transverse	15	IIa	SSA (none)
22	64	F	Ascending	15	Ip	TA (low)
23	56	M	Ascending	15	Ip	TA (low)
25	27	M	Ascending	15	Ip	TVA (high)
35	69	F	Sigmoid	15	Ip	TVA (low)
36	58	F	Ascending	15	Ip	TVA (low)
1b *	75	F	Transverse	20	IIa	VA (high)
2b *	72	F	Transverse	20	IIa	TA (low)
6	79	F	Ascending	20	IIa	TA (low)
7	67	F	Ascending	20	IIa	TA (low)
24	79	M	Ascending	20	Ip	TVA (low)
29	74	F	Sigmoid	20	Ip	TVA (low)
33	58	F	Descending	20	Ip	TA (low)
17b *	54	F	Ascending	20	IIa	TA (low)
**Late-stage (LPLs, diameters: > 20 mm)**						
10	83	M	Ascending	25	IIa	TA (low)
11	66	M	Cecum	25	IIa	TA (high)
18	72	M	Ascending	25	IIa	TA (low)
19a *	79	M	Ascending	25	IIa	VA (low)
20	47	M	Ascending	25	IIa-IIc	MVSP (none)
2a *	72	F	Cecum	30	IIa	TA (low)
26	40	F	Sigmoid	30	Ip	TVA (low)
28	50	M	Sigmoid	30	Ip	TVA (high)
31	69	M	Sigmoid	30	Ip	TA (low)
32	56	M	Sigmoid	30	Ip	TA (low)
34	52	F	Sigmoid	30	Ip	TA (low)
19b *	79	M	Transverse	30	Ip	VA (low)
37	73	M	Ascending	30	Ip	TVA (high)
5	44	M	Hepatic flexure	35	IIa	TA (low)
12	79	M	Ascending	35	IIa-IIc	TVA (high)
3	75	F	Transverse	40	IIa-IIc	TA (high)
4	73	F	Ascending	40	IIa-IIc	SA (high)
9	69	F	Ascending	40	IIa	TVA (low)
30	69	M	Rectum	40	Ip	TA (low)
13a *	54	F	Cecum	45	IIa	TVA (low)
14	74	F	Cecum	50	IIa	TVA (low)
17a *	54	F	Cecum	50	IIa	TA (high)
1a *	75	F	Transverse	70	IIa-IIb	VA (high)
**Invasive lesions (CRCs)**						
**Patient**	**Age**	**Sex**	**Colon segment**	**Stage**^**§**^		**Histologic grade**^**§**^
38	58	F	Ascending	T3N0		G2
39	81	M	Transverse	T2N0		G2
40	61	M	Sigmoid	T3N1		G2
41	69	F	Descending	T4N2		G3
42	77	M	Sigmoid	T2N0		G2
43	67	M	Sigmoid	T3N2		G2
44	67	M	Sigmoid	T3N1		G2
45	57	M	Sigmoid	T3N0		G2
46	81	F	Sigmoid	T2N0		G2
47	77	M	Descending	T3N1		G2
48	73	F	Cecum	T3N1		G3
49	57	M	Sigmoid	T3N0		G2
50	55	M	Descending	T3N0		G2
51	90	F	Cecum	T3N0		G2
52	80	F	Ascending	T3N1		G2
53	75	F	Ascending	T3N0		G2
54	77	F	Cecum	T3N0		G2

* Two lesions were analyzed from this patient.

**†** Paris Endoscopic Classification of Superficial Neoplastic Lesions (Gastrointest Endoscopy 2003;58[suppl.]:S3-S27).

**‡** Preinvasive lesions were classified as tubular adenomas (TA), tubulovillous adenomas (TVA), villous adenomas (VA), microvescicular serrated polyps (MVSP), serrated adenomas (SA), sessile serrated adenomas (SSA). In parentheses, the degree of dysplasia (none, low, high) is reported based on the WHO classification of tumors of the digestive system (Editorial and consensus conference in Lyon, France, November 6–9, 1999 [IARC]).

**§** Sobin LH, Wittekind C. TNM classification of malignant tumours. 6th ed. New York, NY: Wiley-Liss, 2002.

The study was carried out according to the principles of the Declaration of Helsinki and was approved by the Ethics Committees of the Italian hospitals where the tissues were collected (*Istituti Ospitalieri*, Cremona, and *Casa Sollievo della Sofferenza*, San Giovanni Rotondo, Italy). Each subject investigated provided written informed consent to collection and analysis of data and publication of the findings.

### Gene sets

Our analyses focused on 880 functional gene sets from the CP-C2 collection in the Molecular Signatures Database (MSigDB), version 3.0 [16]. These canonical representations of biological pathways or processes have been compiled by domain experts and curated from several online databases (BioCarta, Gene Arrays, BioScience Corp, KEGG, Reactome, Sigma-Aldrich Pathways, Signal Transduction Knowledge Environment, Signaling Gateway).

### Statistical methods

The RS method was used to identify tumor-associated pathway enrichment. In brief, a pathway-level statistic is used to average differential-expression evidence across all genes (e.g., gene-level scores) in a given pathway (gene set C containing *n* distinct genes). The enrichment of pathway C for differentially expressed genes is then measured by comparing C with other hypothetical gene sets made up of the same number (*n*) of genes randomly selected from the array. RS analysis can be used with a variety of gene-level scores. In this case, we used the rank of two-sample *t*-test values of genes in the array [13,14]. The mean and variance of the RS score distribution can be analytically derived, and the induced distribution is approximately Gaussian. This offers an easily computed standardized statistic for measuring pathway enrichment. The RS method has several practical advantages, including high computation efficiency [14], an extremely important feature when large numbers of experiments have to be performed.

For each gene set considered in our analysis, the distribution of the component gene expression levels in the N data subset was independently compared with that of each of the stage-specific tumor subsets (i.e., N vs. SPL, N vs. LPL, and N vs. CRC). In each case, the difference was calculated to quantify tumor-related upregulation or downregulation of the pathway (reflected by positive and negative RS scores, respectively) at that stage of tumorigenesis.

The statistical significance of the RS enrichment score was assessed with non-parametric permutation tests [19]. For this purpose, we computed the nominal p-value of each score by comparing the actual score with the empirical probability density function under the null hypothesis (no genotype-phenotype association) derived using 1000 permutations of the phenotypic labels (0=N, 1=tumor, i.e., SPL, LPL, or CRC lesions). A p-value cut-off of 0.05 was used to define significant pathway enrichment.

Expression data for genes in the Biocarta cell cycle pathway were also subjected to hierarchical clustering analysis and PCA to confirm the relevance of our results. For the former analysis, a Euclidean distance metric and inner squared distance linkage were used to generate hierarchical trees. We analyzed three multi-dimensional data sets, each representing normal mucosa and a given stage of tumor, and clustered heat maps were shown. PCA was applied to the entire multi-dimensional data set representing normal mucosa and tumors of all stages. Each tissue sample was then projected onto the first two principal components to create a 2-dimensional map of the data set.

The validation procedure involved the use of standard GSEA [16], and p-values for the enrichment scores were computed on the basis of 1000 label permutations.

## Results and discussion

**Figure 1:**
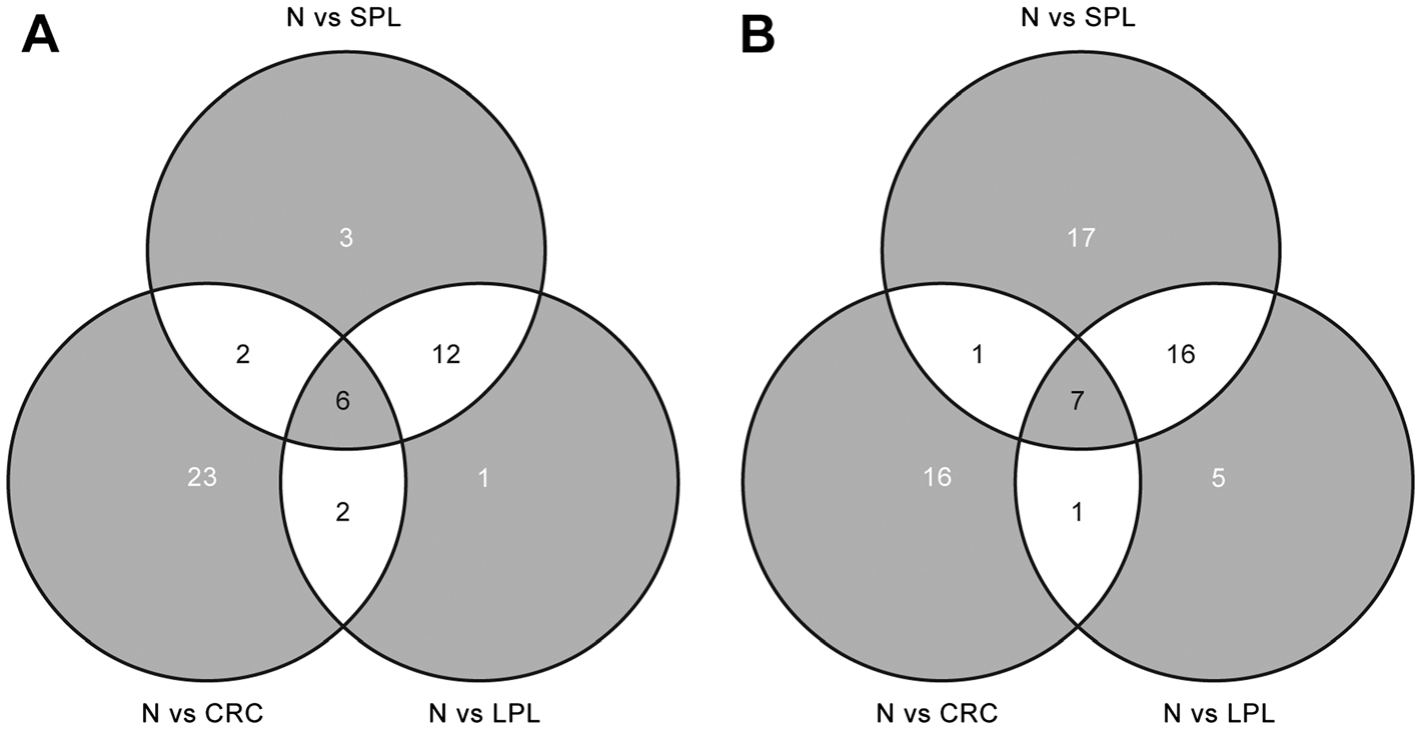
**Numbers of pathways displaying tumor-associated dysregulation at one or more stages of colorectal tumorigenesis.** Venn diagrams show the numbers of pathways that were significantly dysregulated―i.e., upregulated (**A**) or downregulated (**B**) with respect to findings in normal mucosa (N)―in small precancerous lesions (SPLs), large precancerous lesions (LPLs), and colorectal carcinomas (CRCs).

As shown in Tables 2 and 3, a total of 64 pathways were found to be significantly upregulated (n=23) or downregulated (n=41) in SPLs; 50 were upregulated (n=21) or downregulated (n=29) in LPLs; and 58 were upregulated (n=33) or downregulated (n=25) in the CRCs. The approach we used allows in-depth exploration of each instance of pathway dysregulation to characterize its evolution across the transformation process. Because this process is progressive, it was not surprising to find significant dysregulation of certain pathways in 2 or even 3 of the tumor stage-specific data sets, but other alterations were more circumscribed (Figure 1). For example, the BIOCARTA CELL CYCLE PATHWAY (Table 2, row 5) is one of the 23 gene sets that displayed significant upregulation only in the CRCs. This gene set comprises 22 genes (32 RefSeqs) encoding cyclins, cyclin-dependent kinases (CDK), cyclin-dependent kinase inhibitors (CDKI), and transcription factors, including E2F1, whose activation governs the G1-to-S phase transition of the cell cycle. The tumor suppressor RB1 (retinoblastoma protein) negatively regulates cell cycling by complexing with E2F1, and this effect is reversed by the phosphorylation of RB1 by cyclin D/CDK4, cyclin D/CDK6, and cyclin E/CDK2, which releases E2F1 from this complex and allows cell cycling to resume. For this reason, specific inhibitors of the cyclin/CDK complexes, such as p15 (CDKN2B), p16 (CDKN2A), p21 (CDKN1A), and p27 (CDKN1B), are also considered tumor suppressors. Dysregulation of this network stemming (for example) from the overexpression of certain cyclins, CDKs, or E2F1 itself, or from the downregulation of certain CDKIs, can lead to uncontrolled cell growth, which favors tumor formation and progression [20-24].

**Table 2:** Biological pathways displaying up-regulation (versus normal mucosa) in SPLs, LPLs, and CRCs

		**Nominal p-values of enrichment scores†**		
**Pathways**	n*	**N vs SPL**	**N vs LPL**	**N vs CRC**
1) KEGG BASE EXCISION REPAIR	48	0.042	-	-
2) KEGG HOMOLOGOUS RECOMBINATION	34	0.043	-	-
3) REACTOME ACTIVATION OF THE PRE REPLICATIVE COMPLEX	35	0.047	-	-
4) REACTOME HOMOLOGOUS RECOMBINATION REPAIR	22	-	0.048	-
5) BIOCARTA CELLCYCLE PATHWAY	32	-	-	0.025
6) BIOCARTA MONOCYTE PATHWAY	20	-	-	0.035
7) BIOCARTA P27 PATHWAY	14	-	-	0.025
8) BIOCARTA RB PATHWAY	20	-	-	0.047
9) BIOCARTA SET PATHWAY	15	-	-	0.034
10) BIOCARTA SKP2E2F PATHWAY	12	-	-	0.014
11) KEGG RNA POLYMERASE	27	-	-	0.04
12) REACTOME AMINO ACID TRANSPORT ACROSS THE PLASMA MEMBRANE	40	-	-	0.027
13) REACTOME CYTOSOLIC TRNA AMINOACYLATION	26	-	-	0.031
14) REACTOME G1 PHASE	17	-	-	0.03
15) REACTOME GLUCOSE TRANSPORT	55	-	-	0.041
16) REACTOME GLYCOLYSIS	27	-	-	0.039
17) REACTOME NEP NS2 INTERACTS WITH THE CELLULAR EXPORT MACHINERY	39	-	-	0.049
18) REACTOME POST CHAPERONIN TUBULIN FOLDING PATHWAY	9	-	-	0.034
19) REACTOME PREFOLDIN MEDIATED TRANSFER OF SUBSTRATE TO CCT TRIC	25	-	-	0.027
20) REACTOME PROSTANOID HORMONES	15	-	-	0.046
21) REACTOME RNA POLYMERASE III CHAIN ELONGATION	12	-	-	0.033
22) REACTOME RNA POLYMERASE III TRANSCRIPTION INITIATION FROM TYPE 2 PROMOTER	21	-	-	0.047
23) REACTOME TAT MEDIATED HIV1 ELONGATION ARREST AND RECOVERY	31	-	-	0.049
24) REACTOME TRNA AMINOACYLATION	34	-	-	0.048
25) REACTOME TRANSPORT OF RIBONUCLEOPROTEINS INTO THE HOST NUCLEUS	40	-	-	0.043
26) REACTOME VPR MEDIATED NUCLEAR IMPORT OF PICS	48	-	-	0.031
27) SA REG CASCADE OF CYCLIN EXPR	18	-	-	0.01
28) BIOCARTA ARF PATHWAY	24	0.033	0.037	-
29) KEGG NUCLEOTIDE EXCISION REPAIR	48	0.031	0.043	-
30) KEGG ONE CARBON POOL BY FOLATE	19	0.004	0.032	-
31) REACTOME DUAL INCISION REACTION IN GG NER	18	0.032	0.025	-
32) REACTOME G2 M TRANSITION	80	0.038	0.035	-
33) REACTOME MITOCHONDRIAL TRNA AMINOACYLATION	11	0.032	0.04	-
34) REACTOME PURINE METABOLISM	42	0.037	0.03	-
35) REACTOME RNA POLYMERASE I CHAIN ELONGATION	29	0.021	0.026	-
36) REACTOME RNA POLYMERASE I PROMOTER ESCAPE	21	0.027	0.014	-
37) REACTOME RNA POLYMERASE I TRANSCRIPTION INITIATION	25	0.013	0.009	-
38) REACTOME RNA POLYMERASE I TRANSCRIPTION TERMINATION	22	0.027	0.013	-
39) REACTOME SNRNP ASSEMBLY	60	0.031	0.029	-
40) REACTOME MRNA DECAY BY 3 TO 5 EXORIBONUCLEASE	11	0.015	-	0.029
41) REACTOME RNA POLYMERASE III TRANSCRIPTION INITIATION	29	0.041	-	0.047
42) REACTOME NUCLEAR IMPORT OF REV PROTEIN	39	-	0.049	0.043
43) REACTOME REV MEDIATED NUCLEAR EXPORT OF HIV1 RNA	41	-	0.043	0.039
44) BIOCARTA PTC1 PATHWAY	13	0.022	0.018	0.028
45) BIOCARTA RANMS PATHWAY	8	0.013	0.024	0.022
46) REACTOME CYCLIN A1 ASSOCIATED EVENTS DURING G2 M TRANSITION	19	0.038	0.034	0.026
47) REACTOME FORMATION OF TUBULIN FOLDING INTERMEDIATES BY CCT TRIC	18	0.044	0.039	0.011
48) REACTOME PURINE RIBONUCLEOSIDE MONOPHOSPHATE BIOSYNTHESIS	13	0	0.004	0.008
49) REACTOME REGULATION OF GLUCOKINASE BY GLUCOKINASE REGULATORY PROTEIN	42	0.044	0.031	0.044

* *n* = number of RefSeqs in the pathway.

† measured by RS analysis; only significant p-values (< 0.05) are shown.

**Table 3:** Biological pathways displaying down-regulation (compared with normal mucosa) in SPLs, LPLs, and CRCs

		**Nominal p-values of enrichment scores†**		
**Pathways**	n*	**N vs SPL**	**N vs LPL**	**N vs CRC**
1) BIOCARTA AT1R PATHWAY	50	0.047	-	-
2) BIOCARTA BIOPEPTIDES PATHWAY	81	0.027	-	-
3)BIOCARTA IL3 PATHWAY	20	0.044	-	-
4) KEGG ALDOSTERONE REGULATED SODIUM REABSORPTION	51	0.029	-	-
5) KEGG CHEMOKINE SIGNALING PATHWAY	216	0.042	-	-
6) KEGG GAP JUNCTION	100	0.039	-	-
7) KEGG MAPK SIGNALING PATHWAY	400	0.047	-	-
8) KEGG VASCULAR SMOOTH MUSCLE CONTRACTION	152	0.037	-	-
9) REACTOME FORMATION OF PLATELET PLUG	236	0.017	-	-
10) REACTOME FRS2 MEDIATED ACTIVATION	23	0.049	-	-
11) REACTOME HEMOSTASIS	348	0.024	-	-
12) REACTOME METABOLISM OF LIPIDS AND LIPOPROTEINS	256	0.046	-	-
13) REACTOME NRAGE SIGNALS DEATH THROUGH JNK	61	0.041	-	-
14) REACTOME PLATELET ACTIVATION	208	0.018	-	-
15) REACTOME RHO GTPASE CYCLE	132	0.046	-	-
16) REACTOME SEMAPHORIN INTERACTIONS	89	0.031	-	-
17) SA PTEN PATHWAY	27	0.027	-	-
18) REACTOME CAM PATHWAY	32	-	0.042	-
19) REACTOME G ALPHA Z SIGNALLING EVENTS	13	-	0.04	-
20) REACTOME G BETA GAMMA SIGNALLING THROUGH PLC BETA	23	-	0.037	-
21) REACTOME G PROTEIN ACTIVATION	34	-	0.02	-
22) REACTOME NEURORANSMITTER RECEPTOR BINDING AND DOWNSTREAM TRANSMISSION IN THE POSTSYNAPTIC CELL	115	-	0.035	-
23) BIOCARTA NUCLEARRS PATHWAY	22	-	-	0.009
24) KEGG ASCORBATE AND ALDARATE METABOLISM	19	-	-	0.008
25) KEGG DRUG METABOLISM CYTOCHROME P450	71	-	-	0.017
26) KEGG DRUG METABOLISM OTHER ENZYMES	51	-	-	0.045
27) KEGG LONG TERM POTENTIATION	94	-	-	0.024
28) KEGG METABOLISM OF XENOBIOTICS BY CYTOCHROME P450	69	-	-	0.018
29) KEGG NICOTINATE AND NICOTINAMIDE METABOLISM	26	-	-	0.047
30) KEGG PENTOSE AND GLUCURONATE INTERCONVERSIONS	24	-	-	0.009
31) KEGG NITROGEN METABOLISM	28	-	-	0.024
32) KEGG RETINOL METABOLISM	59	-	-	0.028
33) KEGG STARCH AND SUCROSE METABOLISM	54	-	-	0.014
34) REACTOME ACTIVATION OF NMDA RECEPTOR UPON GLUTAMATE BINDING AND POSTSYNAPTIC EVENTS	62	-	-	0.049
35) REACTOME ETHANOL OXIDATION	8	-	-	0.012
36) REACTOME GLUCURONIDATION	15	-	-	0
37) REACTOME MITOCHONDRIAL FATTY ACID BETA OXIDATION	9	-	-	0.039
38) REACTOME PHASE II CONJUGATION	62	-	-	0.036
39) BIOCARTA HDAC PATHWAY	44	0.022	0.041	-
40) KEGG GLYCOSPHINGOLIPID BIOSYNTHESIS LACTO AND NEOLACTO SERIES	37	0.041	0.037	-
41) REACTOME ACTIVATION OF KAINATE RECEPTORS UPON GLUTAMATE BINDING	37	0.022	0.01	-
42) REACTOME ADP SIGNALLING THROUGH P2Y PURINOCEPTOR 1	31	0.01	0.01	-
43) REACTOME ADP SIGNALLING THROUGH P2Y PURINOCEPTOR 12	24	0.028	0.021	-
44) REACTOME GLUCAGON SIGNALING IN METABOLIC REGULATION	42	0.046	0.028	-
45) REACTOME GLUCAGON TYPE LIGAND RECEPTORS	39	0.042	0.036	-
46) REACTOME GS ALPHA MEDIATED EVENTS IN GLUCAGON SIGNALLING	30	0.015	0.012	-
47) REACTOME G BETA GAMMA SIGNALLING THROUGH PI3KGAMMA	30	0.034	0.027	-
48) REACTOME HORMONE SENSITIVE LIPASE HSL MEDIATED TRIACYLGLYCEROL HYDROLYSIS	18	0.045	0.042	-
49) REACTOME IONOTROPIC ACTIVITY OF KAINATE RECEPTORS	14	0.049	0.018	-
50) REACTOME OTHER SEMAPHORIN INTERACTIONS	25	0.005	0.005	-
51) REACTOME PLATELET ACTIVATION TRIGGERS	73	0.028	0.047	-
52) REACTOME SIGNAL AMPLIFICATION	39	0.007	0.005	-
53) REACTOME THROMBIN SIGNALLING THROUGH PROTEINASE ACTIVATED RECEPTORS	28	0.019	0.016	-
54) REACTOME THROMBOXANE SIGNALLING THROUGH TP RECEPTOR	26	0.007	0.003	-
55) KEGG GNRH SIGNALING PATHWAY	150	0.017	-	0.008
56) BIOCARTA STATHMIN PATHWAY	34	-	0.034	0.009
57) BIOCARTA PGC1A PATHWAY	37	0.005	0.011	0.008
58) KEGG PPAR SIGNALING PATHWAY	86	0.026	0.034	0.044
59) KEGG PROXIMAL TUBULE BICARBONATE RECLAMATION	25	0.003	0.003	0.011
60) KEGG SULFUR METABOLISM	18	0.047	0.03	0.038
61) REACTOME NUCLEAR RECEPTOR TRANSCRIPTION PATHWAY	78	0.005	0.006	0.006
62) REACTOME NUCLEOTIDE LIKE PURINERGIC RECEPTORS	23	0.011	0.012	0.022
63) REACTOME P2Y RECEPTORS	18	0.009	0.01	0.036

* *n* = number of RefSeqs in the pathway.

† measured by RS analysis; only significant p-values (< 0.05) are shown.

**Figure 2:**
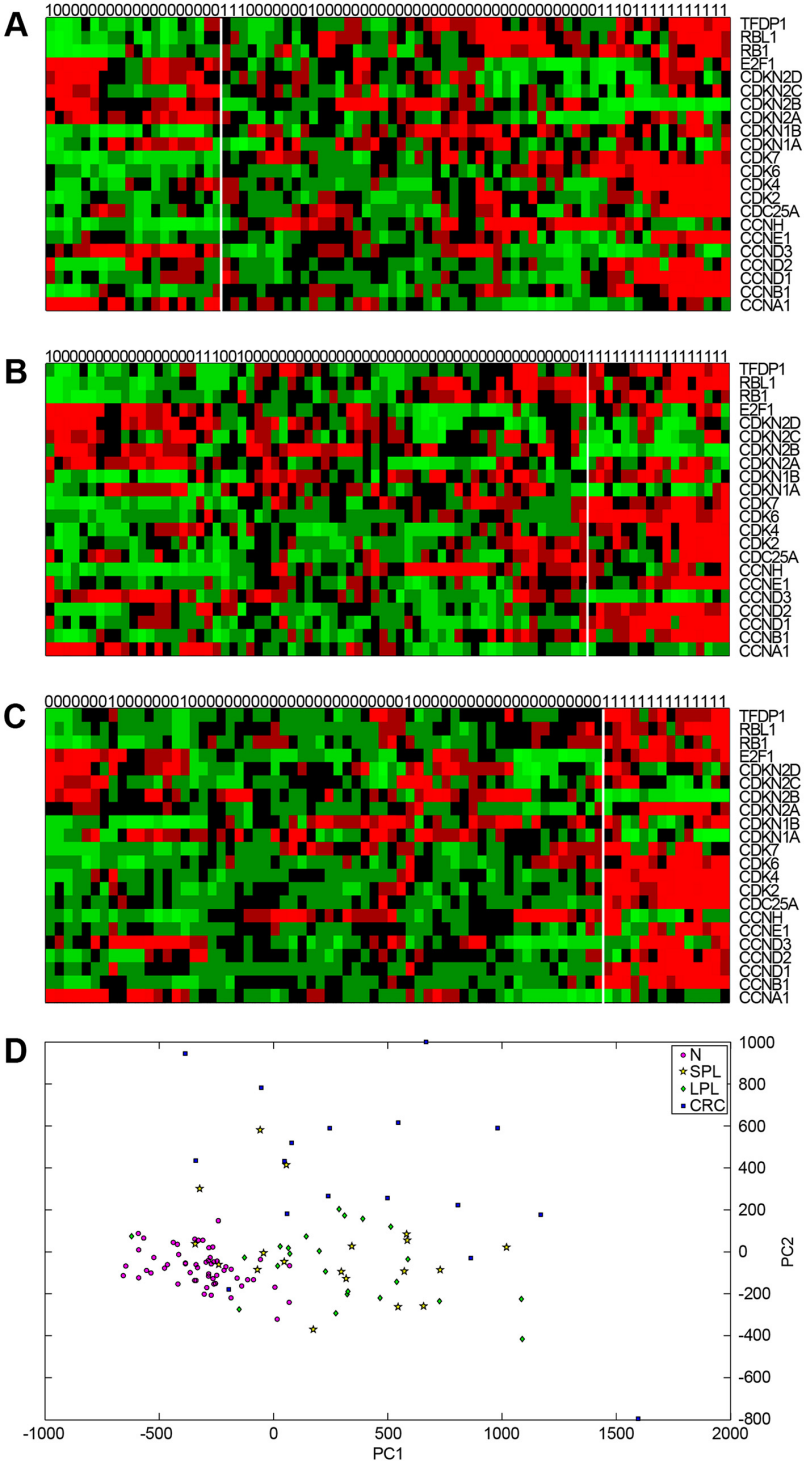
**Hierarchical clustering and PCA of data sets based on cell cycle gene expression.** Heat maps in panels **A**, **B**, and **C** show expression levels for the Biocarta cell cycle pathway’s 22 gene components (listed on the right) across samples in the 3 tumor-stage-specific data subsets: SPLs, LPLs, and CRCs, respectively (each containing corresponding samples of normal mucosa, N). Actual sample labels are shown at the top of each heat map (0=normal mucosa; 1=tumor); the groups identified by hierarchical clustering analysis are separated by vertical white lines. (Dendrograms are not shown.) (**D**) Bi-dimensional projection via PCA of all tumors and normal mucosal specimens using expression levels for the 22 cell cycle-related genes. Each dot represents a tissue sample (pink circle: N; yellow star: SPL; green diamond: LPL; blue square: CRC). The first two components, PC1 and PC2, account for 81% of the variance in this set.

Figure 2 (panels A, B, C) shows heat maps of the expression of the 22 genes included in the Biocarta cell cycle pathway at each stage of tumorigenesis (compared with normal mucosa). Each of the three tumor + N data sets was subjected to hierarchical clustering analysis using the 22 cell cycle-associated genes. As shown in Figure 2A, this analysis identified two clusters within the N vs. SPL data set, which showed no relation to the actual tissue labels (see column labels in Figure 2A). In the N vs. LPL data set (Figure 2B), the two tissue-type groups were more readily distinguished (only 6 LPL samples were misclassified), and in the N vs. CRC set, the two classes of tissues were separated with only three errors. Collectively, these findings point to progressive dysregulation of the cell cycle pathway, which becomes overt in the invasive stage of tumorigenesis, as highlighted by our RS analysis. Major involvement of this pathway at the CRC stage also emerged when the gene expression profiles were subjected to PCA (Figure 2D).

**Figure 3:**
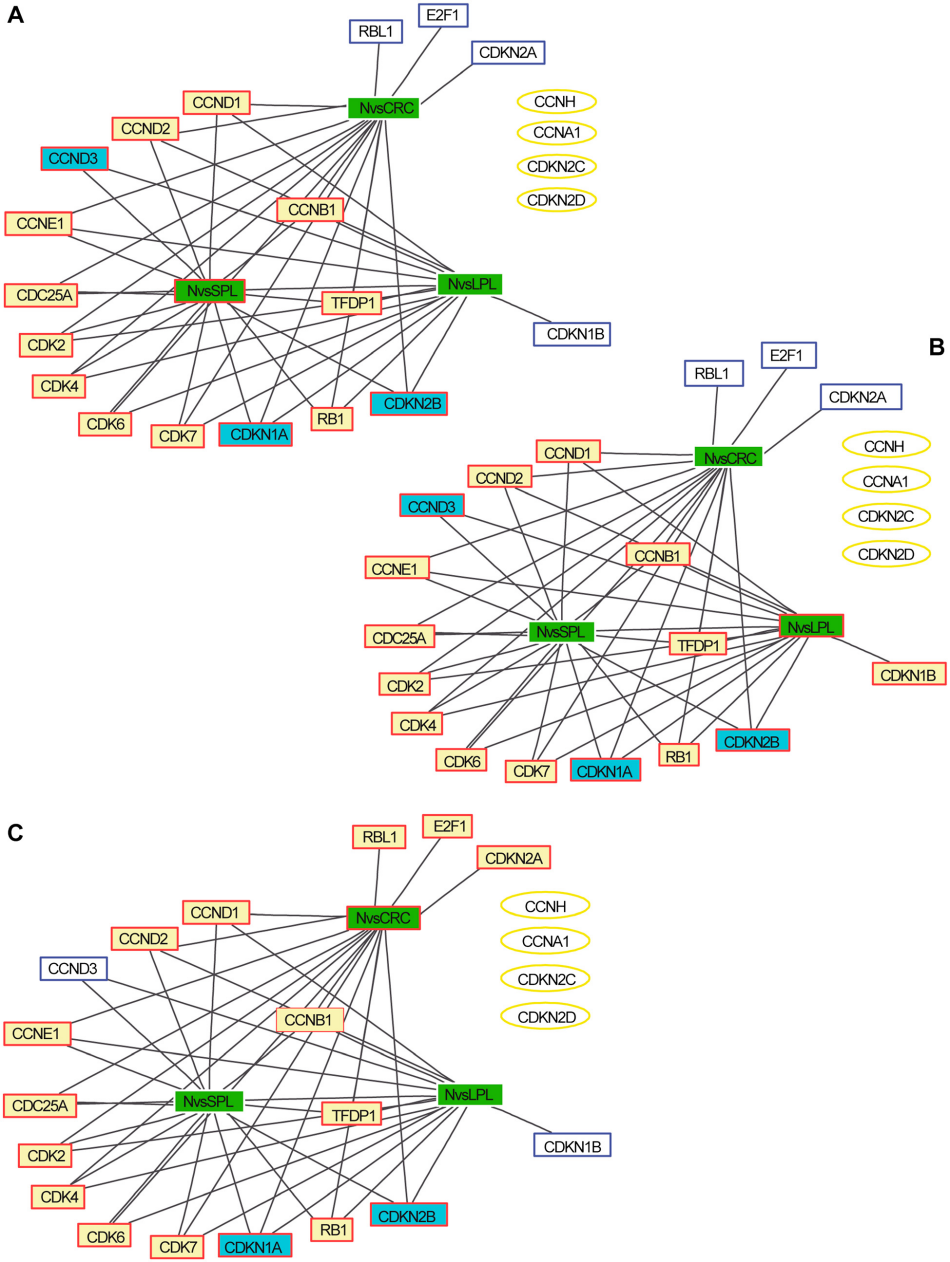
**Dysregulation of the cell cycle pathway during tumor progression.** Expression levels for the 22 Biocarta cell cycle genes in each tumor stage-specific data subset―SPLs (**A**), LPLs (**B**), and CRCs (**C**)―were compared with those in the normal mucosa (N) data set using two-sample *t*-test. Each graph contains 22 nodes representing the genes in the pathway (white, yellow, and blue rectangles, and yellow ellipses) plus a node for each tumor-stage being analyzed (*green rectangles*; those outlined in red represent the stage considered in the panel). *Yellow and blue rectangles*: genes displaying tumor-associated upregulation or downregulation, respectively, in the stage represented in the panel; *white rectangles*: genes that were also dysregulated in at least one of the other two stages; *yellow ellipses:* cell-cycle genes that displayed *no* tumor-related dysregulation at any of the three stages. The connection matrix used for the graph was a sparse square matrix of order 25 where 1 indicates connection between nodes and 0 indicates no connection. *Black lines*: connection between a gene node and tumor-stage node (i.e., tumor-related up- or downregulation of the gene at that stage).

As shown in Figures 3A and 3B, certain cell cycle genes were already overexpressed in SPLs and LPLs, including those encoding CCND1, CCND2, and CCNE1, CDKs 2, 4, 6, and 7, and the oncogenes *CDC25A* and *TFDP1*. These changes were associated with downregulated transcription of the genes encoding the CDKI p15 (CDKN2B) and p21 (CDKN1A), an expected finding for preinvasive lesions with high proliferation rates. In contrast, CDKI p27 (CDKN1B) expression was upregulated in LPLs, but not CRCs (Figure 3C), a finding that is consistent with previously reported immunostaining profiles of adenomatous and cancerous colorectal tissues [25]. Interestingly, the tumor suppressor RB1 was also upregulated across all stages of tumorigenesis (Figure 3), whereas, in previous studies, this alteration has been documented only in the malignant phases [26-28]. The most convincing explanation proposed for the upregulated expression of RB1 and p27 envisions these factors as possible mediators of a homeostatic mechanism that protects cells from the putatively toxic effects of excessive cyclin, CDK, or E2F1 activity [25,28].

One of the most dramatic changes that characterized the transition to CRC (Figure 3C) was an increase in the expression of E2F1, the master regulator of the cell cycle pathway. This alteration is well known in colorectal carcinomas [22,29], and it seems to be associated with higher tumor stages and poorer prognoses in these cancers [30] and those of other organs as well [31-33]. Two other important cell cycle genes, those encoding the tumor suppressors p16 (CDKN2A) and the RB homolog p107 (RBL1), were also upregulated in CRCs. The expression of p16 can be silenced during tumorigenesis by gene promoter methylation, but this phenomenon is largely confined to colorectal cancers with the hypermethylator phenotype and DNA mismatch repair defects, which account for < 20% of all colorectal cancers [34-36]. We have found p16 overexpression in ~80% of the colorectal cancers we have studied over the years (unpublished data). Like the p27 and RB1 upregulation mentioned above (or that of RBL1, which exerts inhibitory effects on E2F1-mediated trans-activation), p16 upregulation might represent a negative feedback mechanism aimed at preventing the G1-to-S transition (although E2F1 can readily overcome a p16-mediated G1 block) [37]. It is interesting to note that the trends shown in Figure 3, which are based on our analysis of transcript levels, are—on the whole—consistent with published data on the corresponding gene products.

Closer inspection of Tables 2 and 3 shows that the pathways exhibiting tumor-related downregulation were generally larger (in terms of the total number of RefSeqs they contained) than those that were upregulated in tumor tissues (mean numbers of RefSeqs in the gene sets: 69 vs. 27.9, respectively; p-value of one-tailed *t*-test = 2.4 · 10^-4^). This finding might be related to the fact that tumor-associated downregulation was often seen in highly conserved pathways that govern normal mucosa homeostasis (e.g., cell differentiation programs). Pathways of this type have been extensively studied since the early days of molecular biology, and a relatively large number of their gene components have been identified. Consequently, the gene sets representing these pathways are likely to be larger than those of more specialized pathways, which have probably been less thoroughly explored. Nonetheless, it is also possible that fundamental pathways and networks are effectively larger as a result of relatively high-level component redundancy, a feature that would increase their robustness and versatility and ensure essential cellular functions in normal tissues under a variety of conditions.

Because the preinvasive stages of colorectal tumorigenesis analyzed in our study have been far less extensively explored than the cancerous phases, there were no independent transcriptomic data sets for precancerous lesions to use to validate our results. To overcome this limitation, we used two different approaches.

First, we re-analyzed our three data sets (N vs. SPL, N vs. LPL, and N vs. CRC) with GSEA [16], in a manner similar to that used in previous studies by our group [13]. Table 4 shows the numbers of pathways displaying significant tumor-associated enrichment in the RS and GSEA analyses. In all cases, a high percentage of the pathways found to be significantly up- or down-regulated in tumors (compared with normal mucosa) displayed the same trend in GSEA. (In both cases, a p-value cut-off of 0.05 was used to define significant enrichment.) For example, in the analysis of N vs. SPL data set, GSEA confirmed the presence of significant tumor-associated enrichment for 21 (91%) of the 23 pathways identified as enriched by our RS analysis (p-values = 0 computed by Fisher’s exact test). The number of enriched pathways identified by GSEA was always substantially higher than that obtained with RS analysis. This finding reflects the fact that in GSEA the nominal p-value of a pathway enrichment score is computed via an empirical phenotype-based permutation test procedure [16]. RS analysis uses a more stringent selection process in which the actual enrichment score of each pathway is compared with the scores obtained by the permutation of labels—an approach similar to that used in GSEA—and with the scores for sets composed of randomly selected genes [17].

**Table 4:** Numbers of pathways displaying significant tumor-associated dysregulation in RS analysis and GSEA of the N vs SPL, N vs LPL, and N vs. CRC data sets

**Differential regulation in tumors**^**†**^	**RS**	**GSEA**	**Overlap**^**‡**^
No. pathways up-regulated in SPLs	23	75	21 (91%)*
No. pathways down-regulated in SPLs	41	121	37 (90%)*
No. pathways up-regulated in LPLs	21	75	20 (95%)*
No. pathways down-regulated in LPLs	29	109	26 (90%)*
No. pathways up-regulated in CRCs	33	52	16 (49%)**
No. pathways down-regulated in CRCs	25	42	21 (84%)*

^†^ No. of pathways found dysregulated by RS and GSEA with p-values < 0.05.

‡ No. (%) of pathways identified as dysregulated in RS analysis that were found to be similarly dysregulated in GSEA; asterisks indicate p-values computed by Fisher’s exact test: *p=0; **p=1.1x10.^-12.^

Second, we validated the findings regarding CRCs by performing RS analysis of two publicly available, independent transcriptomic data sets. The first (V-set I) had been generated by Affymetrix HGU133A GeneChip analysis of 47 samples of human colorectal tissues (22 of normal mucosa, 25 CRCs) and is accessible through the ArrayExpress site (E-MTAB-57). The second (V-set II) was obtained with GeneChip Human Exon 1.0 ST array analysis of 20 paired CRC-normal mucosa samples [38]. The results of these validation analyses are shown in Table 5. The vast majority of pathways exhibiting CRC-related upregulation in the original N vs. CRC data set were also significantly upregulated in V-set I (73%, p-value = 1.1x10^-16^, Fisher’s exact test) and V-set II (82%, p-value = 3.3x10^-16^, Fisher’s exact test). Lower but still excellent degrees of overlap were also observed for the pathways found to be downregulated in CRCs compared with normal mucosa.

**Table 5:** Numbers of pathways displaying significant tumor-associated dysregulation in RS analysis of the N vs CRC data set and in independent validation data sets I and II

**Differential regulation in CRCs**^**†**^	**N vs CRC**	**V-set I**	**V-set II**	**Overlap‡**	
				**N vs CRC and V-set I**	**N vs CRC and V-set II**
No. upregulated pathways	33	107	157	24 (73%) - *1.1x10^-16^	27 (82%) - *3.3x10^-16^
No. downregulated pathways	25	73	58	14 (56%) - *4.6x10^-10^	9 (36%) - *1.1x10^-8^

^†^ No. of pathways found dysregulated by RS with p-values < 0.05.

‡ No. (%) of pathways dysregulated in N vs. CRC data set that were similarly dysregulated in the indicated V-set.

* p-values computed by Fisher’s exact test.

**Figure 4:**
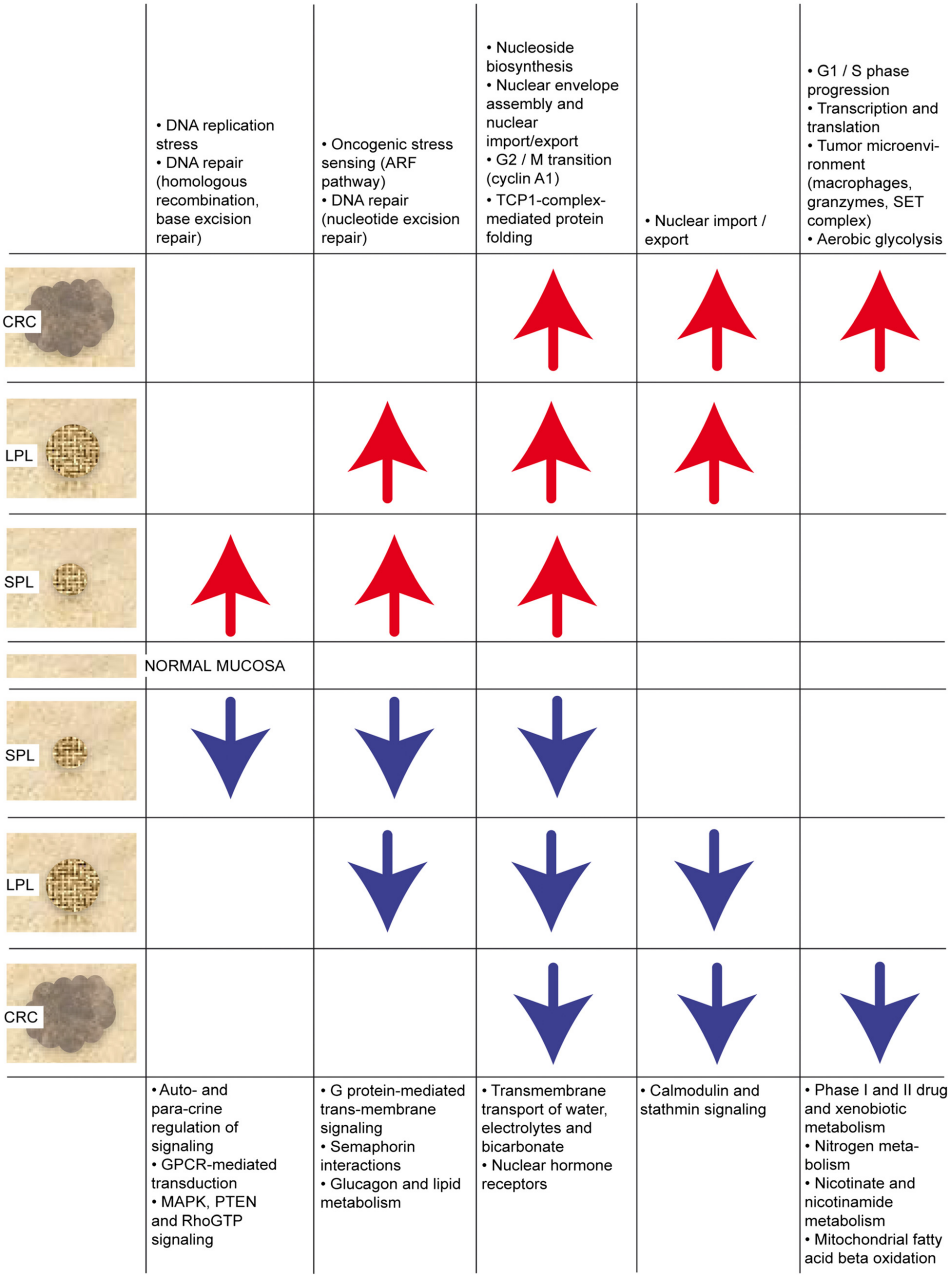
**Overview of tumor-related pathway dysregulation at different stages of transformation.** Pathways displaying identical configurations of dysregulation (e.g., upregulated in SPLs and LPLs but not CRCs) have been combined into 10 more general biological groups (white boxes). Arrows indicate type (up vs. down) of dysregulation.

Figure 4 summarizes the most relevant tumor-related pathway dysregulations at different stages of transformation. Due to space constraints, only the upregulated pathways (Table 2) are discussed below; those that were downregulated (Table 3) are considered in detail in Additional file 1.

Our data suggest that the early preinvasive phase of colorectal tumorigenesis is characterized on the whole by upregulated activity of pathways involved in DNA replication and repair (i.e., KEGG BASE EXCISION REPAIR, KEGG HOMOLOGOUS RECOMBINATION, REACTOME ACTIVATION OF THE PRE-REPLICATIVE COMPLEX). These findings are consistent with recent reports [39,40] showing that the progression of early precancerous lesions (in the colon and elsewhere) is curbed by cell cycle checkpoints that are activated by DNA replication “stress.” The precise nature of this stress is currently unclear, but it is probably initiated by increased expression of or gain-of-function mutations involving oncogenes (e.g., *CCN1*, *KRAS,* or *MYC*), which are known to be early events in tumorigenesis. Abnormal activation of the prereplicative complex entails upregulation of *CDC6* and several minichromosome maintenance genes. (Our data and those described by Freeman et al. [41] might reflect an early step in this type of replicative stress.) This process is associated with stalling and/or collapse of replication forks and double-strand breaks, which slow or arrest the cell cycle to allow the DNA to be repaired (e.g., via homologous recombination). Activation of base excision repair suggests that DNA base oxidation or deamination may also be accelerated in early preinvasive lesions. Paradoxically, each of these repair processes can per se cause genomic instability [40,42]. This would favor the onset and selection of loss-of-function mutations involving tumor suppressor genes, whose protein products drive the cell cycle checkpoints (e.g., *TP53*, which is often mutated in the later phases of colorectal tumorigenesis [1]), and the result would be unrestrained tumor progression.

In line with the above findings, two other pathways also appeared to be upregulated in our SPLs and LPLs. The BIOCARTA ARF PATHWAY emanates from the tumor suppressor proteins p16INK4a and p14ARF (both encoded by *CDKN2A*). It is a key sensor of oncogenic stress (e.g., the *KRAS*- or *MYC*-associated hyperproliferative signal documented in colorectal adenomas). Activation of the ARF pathway stabilizes TP53, thereby promoting effective checkpoint activity [43]. Both classes of preinvasive lesions also displayed upregulated nucleotide excision repair (KEGG NUCLEOTIDE EXCISION REPAIR), which targets UV- and carcinogen-induced DNA adducts [44]. In conditions of replicative stress, sustained activation of this pathway might be triggered by the complex (but poorly defined) mixture of putative carcinogens generated in the colorectum by host and bacterial metabolism.

DNA damage checkpoints and apoptosis appear to be efficient barriers that can restrain tumor growth for up to two decades [45]. Nonetheless, DNA replication stress and repair are naturally associated with increased cell proliferation rates in colorectal tumors. The need for DNA building blocks, before and after these barriers have been disrupted, explains why nucleotide metabolism is increased throughout tumorigenesis, as reflected by the early persistent upregulation we observed in the REACTOME PURINE RIBONUCLEOSIDE MONOPHOSPHATE BIOSYNTHESIS pathway and also by that of the KEGG PYRIMIDINE METABOLISM pathway. (The significance of the latter upregulation was borderline, so it is not listed in Table 2.)

DNA replication is followed by dramatic changes in the nucleus and its membrane during mitosis, so it was not surprising that the RAN/mitotic spindle pathway (BIOCARTA RANMS PATHWAY) was upregulated at all three stages of tumorigenesis. The small nuclear GTPase RAN (ras-related nuclear protein) directs the assembly of the mitotic spindle and later that of the nuclear envelope, whose nuclear pore complexes are necessary to re-establish nucleocytoplasmic transport [46]. Pathways involved in the G2-to-M transition of the cell cycle (e.g., REACTOME CYCLIN A1 ASSOCIATED EVENTS DURING G2 M TRANSITION) were also constantly upregulated during tumorigenesis, as was the REACTOME FORMATION OF TUBULIN FOLDING INTERMEDIATES BY CCT TRIC pathway, which is involved in protein folding mediated by the chaperonin containing the TCP1 complex. This complex plays central roles in the folding and assembly of numerous proteins [47], so the upregulated expression of several genes encoding its subunits could be easily ascribed to increased protein metabolism in tumor cells.

Of the 23 pathways selectively upregulated in CRCs, six pointed to the activation of the G1-to-S phase transition: SA REG CASCADE OF CYCLIN EXPR (Regulatory cascades of cyclin expression), BIOCARTA SKP2E2F PATHWAY, BIOCARTA CELLCYCLE PATHWAY, BIOCARTA P27 PATHWAY, REACTOME G1 PHASE, and BIOCARTA RB PATHWAY (see also first section of *Results and Discussion*). The simultaneous upregulation of these inter-related cell-cycle pathways in advanced colorectal tumors reflects the sustained proliferation that is a fundamental trait of cancer cells [48]. The invasive stages of tumorigenesis are thought to be characterized by mutations involving tumor suppressor genes like *TP53* or *PTEN*, alterations that allow cancer cells to circumvent programs that limit proliferation (e.g., the cell-cycle checkpoints, which operate more efficiently in early-stage tumors, as discussed above). This high-proliferation environment is naturally associated with increased transcription and translation, as documented in our dataset by the upregulation of diverse RNA polymerase II and III functions, amino-acid transport across the plasma membrane, and tRNA aminoacylation (Table 2).

Over the past 20 years, important roles have emerged for nonepithelial cells in the progression of colorectal adenocarcinomas (and those involving other organs) [48]. Macrophages, for example, seem to play conflicting (but nonetheless crucial) roles in both tumor development and metastasis [49], and this is consistent with the marked upregulation of the BIOCARTA MONOCYTE PATHWAY observed in our CRC dataset. Monocyte differentiation gives rise to tumor-antagonizing and tumor-promoting macrophages. The latter cells promote angiogenesis, enhance tumor cell migration and invasion, and suppress antitumor immunity [49]. CRC-related upregulation of the BIOCARTA SET PATHWAY reflects the importance of another stromal contribution to colorectal carcinogenesis: granzyme release by cytotoxic T lymphocytes. These serine proteases (along with the multiprotein SET complex, whose components are encoded by genes frequently upregulated in our tumors) trigger apoptosis and are therefore regarded as mediators of antitumor immunity [50]. But they can also provoke inflammation and cleave extracellular matrix components [50]. Moreover, the SET protein is believed to act as an oncoprotein (given its apoptosis-inhibiting activity within the SET complex) and as a regulator of chromatin remodeling [51,52]. On the basis of our transcriptomic data alone, it is difficult to discern what type of impact SET pathway activation has on colorectal cancer progression.

Finally, the REACTOME GLYCOLYSIS pathway was found to be upregulated in CRCs. Since its first description in 1924 by Otto Warburg [53], aerobic glycolysis has been considered the preferred pathway for metabolizing glucose in cancer cells (as opposed to the oxidative metabolism used by normal differentiated cells). Our data demonstrate that the switch to aerobic metabolism can be documented with transcriptional analysis of the genes encoding metabolic enzymes. Cancer cells appear to exploit aerobic glycolysis to produce the biomass needed for new cells, despite the pathway’s inefficient ATP generation [54]. Cancer cells’ need for nutrients to fuel biomass production is also reflected in the activation of other pathways mentioned above, such as those involving glucose and amino-acid transport, regulation of glucokinase, and purine biosynthesis.

## Conclusions

Our exhaustive description of the sequence of critical molecular events characterizing the progression of colorectal tumors is based on a statistically robust analysis of transcriptomic data carried out at the level of functional molecular processes rather than individual genes or proteins. This analysis revealed specific pathways whose dysregulation might play a role in each transition of the transformation process. This is the first study in which such an approach has been used to gain further insights into colorectal tumorigenesis. Therefore, our findings provide a foundation for larger projects in which transcriptomic data will be integrated with (epi)genomic, proteomic, and metabolomic data from ongoing and future studies. They should open roads to experimental research aimed at providing more in-depth, systems-level understanding of colorectal tumorigenesis.

## Abbreviations

GSEA: Gene Set Enrichment Analysis; RS: Random Set; CRC: colorectal cancer; SPL: small preinvasive lesion; LPL: large preinvasive lesion; N: normal mucosa; MSigDB: Molecular Signatures Database; PCA: Principal Component Analysis.

## Competing interests

The authors declare that they have no competing interests.

## Authors’ contributions

GM, NA, AA conceived the study. RM performed the statistical analysis and together with VCL and EL compared the experimental results. EC, MC, OP, AP, APa, TS, FB were mainly involved in population study, RNA extraction and they provided the final DNA microarray data set. All the authors contributed to the drafting of the article. All authors read and approved the final manuscript.

## Pre-publication history

The pre-publication history for this paper can be accessed here:

http://www.biomedcentral.com/1471-2407/12/608/prepub

## Supplementary Material

Additional file 1 **Biological pathways found to be downregulated at different stages of colorectal transformation (Table **3**and Figure **4**).** Click here for file
