# Supplementary material for: Molecular pathways undergoing dramatic transcriptomic changes during tumor development in the human colon
Source: BMC Cancer. 2012 Dec 19;12:608. doi: 10.1186/1471-2407-12-608 (PMC3541196; doi:10.1186/1471-2407-12-608)
Supplement: Additional file 1 — Biological pathways found to be downregulated at different stages of colorectal transformation (Table 3and Figure 4). [file 1471-2407-12-608-S1.doc]

## Additional file 1

## Biological pathways found to be downregulated at different stages of colorectal transformation (Table 3 and Figure 4)

Seventeen pathways displayed over-representation of genes that were downregulated in SPLs (Table 3 and Figure 4), but there was substantial overlap among the genes belonging to 13 of these gene sets. Most of the downregulated genes encode proteins involved in early-phase signal transduction mediated by plasma membrane proteins (usually G-protein-coupled receptors [1,2], which activate interconnected cytoplasmic cascades (such as the MAPK, PTEN, and Rho GTPase signaling; i.e., KEGG MAPK SIGNALING PATHWAY, SA PTEN PATHWAY and REACTOME RHO GTPASE CYCLE) that can exert profound effects on phosphorylation / dephosphorylation processes and calcium / calmodulin signaling [3,4,5,6]. The overall effect of this attenuated signaling in the early stages of colorectal tumorigenesis is difficult to identify. For example, downregulation of the PTEN pathway would sustain tumorigenesis [7], but diminished signaling through the MAPK cascades would have the opposite effect [8]. Functional studies on individual branches of these pathways and their interconnections are necessary for a more accurate interpretation of our transcriptomic data. Most of these signal transduction pathways are affected by interactions between epithelial and stromal cells, and some pathways involved in these interactions are also downregulated in SPLs (for example, BIOCARTA BIOPEPTIDES PATHWAY, KEGG CHEMOKINE SIGNALING PATHWAY, REACTOME SEMAPHORIN INTERACTIONS, KEGG VASCULAR SMOOTH MUSCLE CONTRACTION, and REACTOME HEMOSTASIS). Qualitative and quantitative differences in the stromal contents of normal and tumor tissues are factors to consider in the interpretation of these data and in planning functional studies.

Downregulation of transmembrane signaling systems was also evident from the analysis of the 16 pathways concomitantly attenuated in both SPLs and LPLs. Key mediators of these systems are G proteins [9]. Many of the genes encoding these proteins (represented in 8 out of 16 pathways shown in Table 3) were significantly down-regulated. G protein signal transduction is a component of various pathways. Those that were most markedly downregulated in our data set are related to hemostasis (above all platelet functions) and glucagon metabolism. Like SPLs (as discussed above), LPLs were also characterized by downregulation of pathways related (in various ways) to the tissue microenvironment (e.g., semaphorin interactions, neurotransmission, myogenesis). The general impression is that the initial transformation of normal mucosa is accompanied by a dramatic drop in basic transmembrane signaling processes that maintain epithelial / stromal homeostasis in the normal colorectal mucosa (including auto- and paracrine regulations). The plasma membrane also appears to undergo radical remodeling, as suggested by the downregulation of genes involved in lipid, lipoprotein, and glycosphingolipid metabolism (e.g., those in the KEGG GLYCOSPHINGOLIPID BIOSYNTHESIS LACTO AND NEOLACTO SERIES and REACTOME HORMONE SENSITIVE LIPASE HSL MEDIATED TRIACYLGLYCEROL HYDROLYSIS gene sets).

Other downregulated pathways point to plasma membrane alterations across all stages of the transformation process (such as the KEGG PROXIMAL TUBULE BICARBONATE RECLAMATION and REACTOME P2Y RECEPTORS). These changes include underexpression of carbonic anhydrases, P2Y receptors, and numerous integral membrane proteins involved in the transmembrane transport of water, electrolytes, and bicarbonate. This finding strongly supports the central role played by loss of cell differentiation in tumorigenesis, and our data indicate that this phenomenon occurs largely at the cell-membrane level. All three tumor stages exhibited striking down-regulation of the REACTOME NUCLEAR RECEPTOR TRANSCRIPTION PATHWAY and KEGG PPAR SIGNALING PATHWAY, which mainly involved genes belonging to the nuclear hormone receptor family (e.g., *NR5A2*, *RORA*, and *PPARG*) [10]. They encode cytoplasmic receptors for lipophilic ligands (xenobiotic or endogenous), such as steroid hormones, vitamin D, retinoids, bile acids, derivatives of dietary lipids, and many others. Ligand binding alters the conformation of these receptors, which interact with other proteins to gain entry to the nucleus, where they regulate the transcription of numerous genes (see below). How the loss of this regulation affects colorectal tumorigenesis is a question that deserves further investigation.

G protein-mediated events in the cytosolic compartment might be subject to additional dysregulation in LPLs and CRCs. These tumors displayed decreased expression of several genes in the adenylate cyclase and cAMP-dependent protein kinase families and others involved in calcium / calmodulin signaling [3,4,5,6] (i.e., BIOCARTA STATHMIN PATHWAY). The invasive stage was characterized by significant suppression of numerous metabolic processes, many of which involve xenobiotics and/or drugs. Phase II conjugation processes [11] (e.g., glucuronidation, which is involved in the metabolism of diverse endogenous and xenobiotic compounds) appeared to be particularly affected, as reflected by the underexpression of a large number of UDG glucuronosyltransferases in the REACTOME GLUCURONIDATION gene set. However, phase I reactions (e.g, KEGG DRUG METABOLISM CYTOCHROME P450) [11,12] were also widely represented among the downregulated metabolic pathways in our data set. The sustained attenuation of nuclear receptor-mediated signaling during colorectal tumorigenesis (see above) would also have an impact on drug metabolism since these receptors are xenosensors and control the expressions of drug-metabolizing enzymes and transporters [11]. This dramatic change in drug metabolism deserves more in-depth exploration, given its potential implications for cancer treatment. The rational use of drugs and bioactive dietary components at this late stage of the disease also requires a sound understanding of the metabolic changes that occur in primary and metastatic cancer cells [13,14].

Our analysis may well have missed certain pathways whose key roles in colorectal tumorigenesis are well-established. A good example is the Wnt signaling pathway. The fact that this gene set displayed no significant enrichment for differentially expressed genes in any of the data sets we analyzed by no means suggests that this pathway is not dysregulated in colorectal tumors. Our data set reflects *transcript-level* changes occurring during tumorigenesis, whereas pathway activity can also be altered by post-transcriptional and/or post-translational mechanisms. Indeed, post-translational modifications of β-catenin (a major component of the Wnt signaling cascade) are known to be responsible for alterations involving the content and subcellular localization of total and active forms of this protein [15].

1. Huang CC, Tesmer JJ**: Recognition in the face of diversity: interactions of heterotrimeric G proteins and G protein-coupled receptor (GPCR) kinases with activated GPCRs.** *J Biol Chem* 2011, **286**: 7715–7721.
2. Gruber CW, Muttenthaler M, Freissmuth M: **Ligand-based peptide design and combinatorial peptide libraries to target G protein-coupled receptors.** *Curr Pharm Des* 2010, **16**: 3071–3088.
3. Haiech J, Audran E, Feve M, Ranjeva R, Kilhoffer MC: **Revisiting intracellular calcium signaling semantics**. *Biochimie*, in press.
4. Wayman GA, Tokumitsu H, Davare MA, Soderling TR: **Analysis of CaM-kinase signaling in cells.** *Cell Calcium,* in press.
5. Parekh AB: **Decoding cytosolic Ca2+ oscillations.** *Trends Biochem Sci* 2011, **36**: 78–87.
6. Parekh AB, Muallem S: **Ca(2+) signalling and gene regulation.** *Cell Calcium,* in press.
7. Hollander MC, Blumenthal GM, Dennis PA: **PTEN loss in the continuum of common cancers, rare syndromes and mouse models.** *Nat Rev Cancer* 2011, **11**: 289–301.
8. Fang JY, Richardson BC: **The MAPK signalling pathways and colorectal cancer.** *Lancet Oncol* 2005, **6**: 322–327.
9. Tesmer JJ: **The quest to understand heterotrimeric G protein signaling.** *Nat Struct Mol Biol* 2010, **17**: 650–652.
10. D'Errico I, Moschetta A: **Nuclear receptors, intestinal architecture and colon cancer: an intriguing link.** *Cell Mol Life Sci* 2008, **65**: 1523–1543.
11. Omiecinski CJ, Vanden Heuvel JP, Perdew GH, Peters JM: **Xenobiotic metabolism, disposition, and regulation by receptors: from biochemical phenomenon to predictors of major toxicities.** *Toxicol Sci* 2011, **120**(Suppl 1): S49–75.
12. Tamasi V, Monostory K, Prough RA, Falus A: **Role of xenobiotic metabolism in cancer: involvement of transcriptional and miRNA regulation of P450s.** *Cell Mol Life Sci* 2011, **68**: 1131–1146.
13. Spratlin JL, Serkova NJ, Eckhardt SG: **Clinical applications of metabolomics in oncology: a review.** *Clin Cancer Res* 2009, **15**: 431–440.
14. Moon YJ, Wang X, Morris ME: **Dietary flavonoids: effects on xenobiotic and carcinogen metabolism.** *Toxicol In Vitro* 2006, **20**: 187–210.
15. Daugherty RL and Gottardi CJ: **Phospho-regulation of Betacatenin adhesion and signaling functions.** *Physiology* 2007, **22:** 303–309.
